# Evaluation of the In Vitro Damage Caused by Lipid Factors on Stem Cells from a Female Rat Model of Type 2 Diabetes/Obesity and Stress Urinary Incontinence

**DOI:** 10.3390/ijms21145045

**Published:** 2020-07-17

**Authors:** Istvan Kovanecz, Robert Gelfand, Sheila Sharifzad, Alec Ohanian, William Brent DeCastro, Carley Cooper, Guiting Lin, Tom Lue, Nestor F. Gonzalez-Cadavid

**Affiliations:** 1 Division of Urology, Department of Surgery, Harbor-UCLA Medical Center and The Lundquist Institute at Harbor-UCLA Medical Center, Torrance, CA 90502, USA; ikovanecz@lundquist.org (I.K.); coastalbob@sbcglobal.net (R.G.); sharifzad.sh@gmail.com (S.S.); alec.ohan@gmail.com (A.O.); williambrentdecastro@cdrewu.edu (W.B.D.); ccooper92@csu.fullerton.edu (C.C.); 2 Department of Urology, David Geffen School of Medicine at UCLA, Los Angeles, CA 90095-1768, USA; 3 Department of Medicine, Charles Drew University of Medicine and Science, Los Angeles, CA 90059, USA; 4 Department of Urology, UCSF School of Medicine, San Francisco, CA 94143, USA; Guiting.Lin@ucsf.edu (G.L.); Tom.Lue@ucsf.edu (T.L.)

**Keywords:** stem cell damage, muscle-derived stem cells, dyslipidemia, cholesterol, palmitic acid, free fatty acids, fat infiltration, apoptosis, wound closure, microRNA, myostatin

## Abstract

Human stem cell therapy for type 2 diabetes/obesity (T2D/O) complications is performed with stem cell autografts, exposed to the noxious T2D/O milieu, often with suboptimal results. We showed in the Obese Zucker (OZ) rat model of T2D/O that when their muscle-derived stem cells (MDSC) were from long-term T2D/O male rats, their repair efficacy for erectile dysfunction was impaired and were imprinted with abnormal gene- and miR-global transcriptional signatures (GTS). The damage was reproduced in vitro by short-term exposure of normal MDSC to dyslipidemic serum, causing altered miR-GTS, fat infiltration, apoptosis, impaired scratch healing, and myostatin overexpression. Similar in vitro alterations occurred with their normal counterparts (ZF4-SC) from the T2D/O rat model for female stress urinary incontinence, and with ZL4-SC from non-T2D/O lean female rats. In the current work we studied the in vitro effects of cholesterol and Na palmitate as lipid factors on ZF4-SC and ZL4-SC. A damage partially resembling the one caused by the female dyslipidemic serum was found, but differing between both lipid factors, so that each one appears to contribute specifically to the stem cell damaging effects of dyslipidemic serum in vitro and T2D/O in vivo, irrespective of gender. These results also confirm the miR-GTS biomarker value for MDSC damage.

## 1. Introduction

The intrinsic assumption in the literature is that stem cells are inherently resistant to any systemic noxious milieu created in vivo by chronic diseases, particularly type 2 diabetes and obesity (T2D/O), and aging and other factors, known to impair and kill differentiated cells in most long-term pathophysiological processes. This view was very rarely challenged conceptually and even less experimentally until recently in terms of finding and/or inducing and characterizing stem cell damage. This also applied for the stem cells endogenous role in tissue repair or their use as autografts in human stem cell therapy, where in T2D/O patients with urological disorders [1,2,3,4] clinical results are often suboptimal. The therapeutic efficacy generally observed in their respective animal models is not often reproduced clinically. 

We tested our hypothesis that the above assumption of stem cell resilience is incorrect, in the male Obese Zucker (OZ) rat model of T2D/O associated with erectile dysfunction [5]. It was shown that muscle-derived stem cells (MDSC) from aged rats, long exposed in vivo to a highly dyslipidemic and moderately hyperglycemic systemic milieu, were imprinted with an abnormal gene global transcriptional signature (gene-GTS). When implanted in vivo into the penile corpora cavernosa of aged OZ rats, these stem cells were severely impaired in their expected correction of erectile dysfunction and the underlying penile corporal pathology. This was associated with overexpression of myostatin, the main inhibitor of muscle mass, and a lipofibrotic effector [6]. The T2D/O milieu induced MDSC damage and the loss of their tissue and functional repair capacity upon corporal implantation [5]. These effects were not seen in the MDSC from young rats with only incipient early T2D/O, implanted as controls. 

Subsequently [7], our group demonstrated that some of these effects could be mimicked in cell culture. This was done by short-term incubation of the male MDSC from young OZ rats (virtually not exposed to T2D/O) with dyslipidemic serum from the aged, very obese, and mild hyperglycemic OZ rats. Obviously, the endogenous stem cells had failed in these aged animals to prevent or reverse erectile dysfunction and the loss of the corporal smooth muscle cells that underlies it. In vitro, this dyslipidemic serum from aged OZ rats caused fat infiltration, apoptosis, myostatin overexpression, and impaired differentiation in the “normal” MDSC. Some changes occurred in vitro in these male MDSC with cholesterol (CHOL), and palmitic acid (PA) as a saturated free fatty acid. 

Some of the in vitro induced gene-GTS alterations by PA and CHOL, but not by dyslipidemic serum, resembled the previously reported in vivo changes. However, their marked in vitro effects (including serum) on the epigenetic microRNA transcription (miR-GTS), similar to the miR changes found in vivo by long-term T2D/O, were the most remarkable alterations. This led us to consider miR-GTS as biomarkers of the in vitro and in vivo MDSC damage. We proposed that they could be used to identify ineffective stem cells, particularly to exclude their use in autologous human stem cell therapy. This was at least for erectile dysfunction in T2D/O patients, if these or other miR alterations are confirmed in human stem cells.

Finally, another study from our group investigated [8] whether this damage induced in vitro on male stem cells may similarly occur in female MDSC involved in endogenous tissue repair, and consequently, the gender dependency of MDSC susceptibility to damage. If the damage was gender independent, it might be used for predicting the impairment of stem cell therapy of another urological condition, female stress urinary incontinence (FSUI), that affects mainly the bladder urethral sphincter [1,2,3,9]. This was relevant to the high prevalence of FSUI in women with T2D/O and the variable degree of efficacy of autograph stem cell therapy for this condition [1,2,3]. Female rats of the same strain as the male OZ rat model (carrying the leptin gene mutation for T2D/O) were used but they were named Zucker fatty or ZF rats to avoid confusion [8]. 

It was shown that a short-term in vitro exposure of MDSC, isolated from the young female ZF rats (ZF4-SC) to highly dyslipidemic serum from the aged diabetic/obese female ZF rats, essentially replicated the fat deposition, apoptosis, and myostatin overexpression previously described for the male MDSC. The same occurred with MDSC isolated from young, non-T2D/O Zucker lean (ZL) rats (ZL4-SC). The inhibition of in vitro scratch wound healing by dyslipidemic serum was similar for both ZF4-SC and ZL4-SC to the one of male MDSC. In terms of miR-GTS, the dyslipidemic ZF serum affected both female stem cells more severely than the male MDSC, indicating some gender-specific susceptibility. It was proposed that some of the changes in miR-GTS might predict in vivo noxious effects of the T2D/O milieu, specifically by dyslipidemia rather than hyperglycemia, which may impair autograft stem cell therapy for FSUI.

In the current study we aimed to find whether cholesterol and Na palmitate, which had induced some in vitro alterations in male rat MDSC during short-term incubations, also led female ZF4-SC and ZL4-SC to a somewhat similar damage, and whether this resembled the one caused by the female dyslipidemic serum. This, in order to further support the assumption that these lipids are effectors, irrespective of gender, of the stem cell damage by dyslipidemia and the T2D/O systemic milieu, and that miR-GTS serve as biomarkers. 

## 2. Results

As previously reported [8], 12 weeks old female Zucker Fatty (ZF) rats (ZUC-Lepr^fa/fa^186), and their Zucker Lean (ZL) controls (ZUC-Lepr^fa^185) of the same strain used for the study on male rats [5,6,7] were used here for isolating the female stem cells. The ZF rats are a model of metabolic syndrome, in the male evolving into frank diabetes and morbid obesity, whereas in the female leading to a more marginal hyperglycemia and moderate obesity, both identified as T2D/O. This female rat model is associated with FSUI detected at 12 [9] to 16 weeks of age [10]. The female MDSC (to differentiate them from the male MDSC) were named ZF4-SC (from the ZF rats) and ZL4-SC (from the ZL control rats). 

We wished to determine whether the effects previously found with both types of female stem cells incubated for 4–5 days with 5% highly dyslipidemic serum from ZF rats aged to over 23 months of age [8], were reproduced with individual serum lipids. The incubations in the current work were performed with water-soluble preparations of cholesterol at 50 mg/dL, or Na palmitate (as representative free fatty acid) at 0.5 mM, instead of dyslipidemic serum addition, for all the tests. 

**Figure 1:**
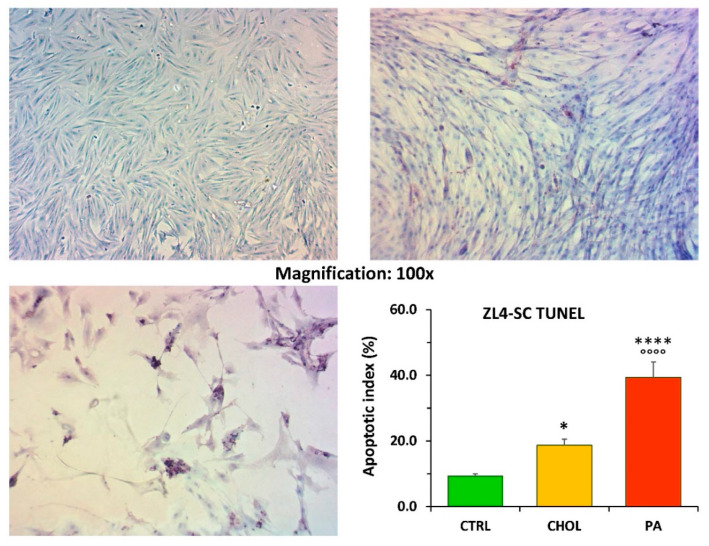
The short-term incubation of ZL4-SC with cholesterol and particularly with Na palmitate (as saturated free fatty acid) stimulated apoptosis. ZL4-SC were incubated at 10% confluence for 4 days in triplicate wells, either without any addition as controls (CTRL), or after adding water-soluble cholesterol (50 mg/dL) (CHOL), or Na palmitate (0.5 mM) and then stained with the TUNEL reaction. Representative pictures are on top left: CTRL; top right: CHOL; and bottom left: palmitic acid (PA). Quantitative image analysis of multiple fields covering each well were performed, counting apoptotic cells, and the means ± SEM apoptotic index were plotted on the bottom right. **** *p* < 0.0001 (PA vs. CTRL); °°°° *p* < 0.0001 (PA vs. CHOL); * *p* < 0.05 (CHOL vs. CTRL).

Figure 1 shows that in ZL4-SC, cholesterol doubled the low apoptotic index found in the control without addition, measured by quantitative image analysis of the TUNEL reaction, resembling what was reported for the dyslipidemic serum added to 5% [8]. Na palmitate was much more damaging, nearly quadruplicating apoptosis.

**Figure 2:**
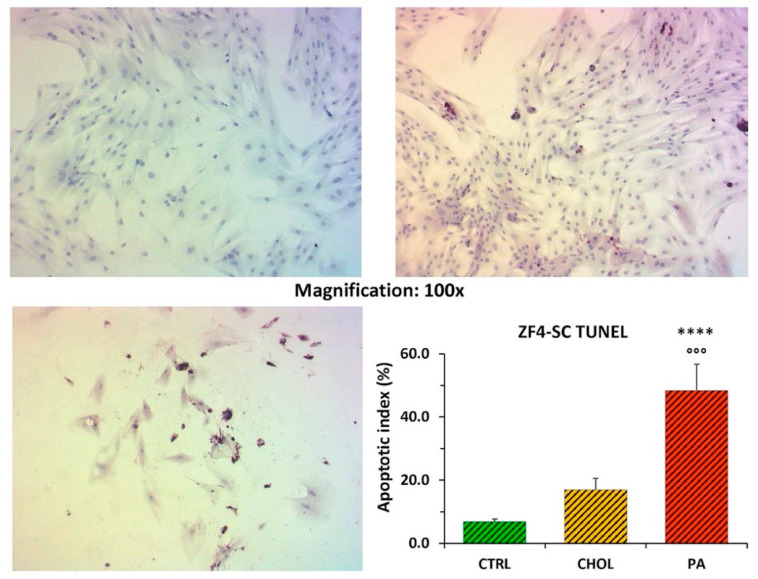
The ZF4-SC were more sensitive than ZL4-SC to apoptosis by incubation with Na palmitate, but less with cholesterol. For description, see legend to Figure 1. **** *p* < 0.0001 (PA vs. CTRL); °°° *p* < 0.001 (PA vs. CHOL). CHOL vs. CTRL was non-significant (NS).

The mild apoptotic effect of cholesterol on ZF4-SC was like on ZL4-SC, but for palmitate its strong effect was slightly exacerbated (Figure 2).

**Figure 3:**
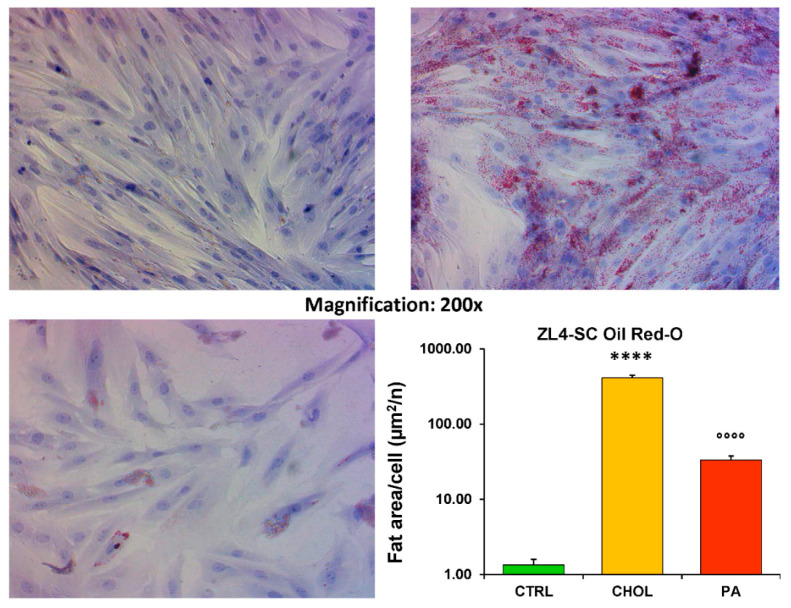
The low apoptosis response of the ZL4-SC to cholesterol was accompanied paradoxically by a considerable fat infiltration much higher than by Na palmitate. ZL4-SC were incubated as depicted in Figure 1, but at completion, the wells were stained for fat droplets by Oil Red O. For the panel distribution and image analysis, see Figure 1, but on the bar graph, the means ± SEM (*n* = 24) fat values are plotted on a logarithmic scale. **** *p* < 0.0001 (CHOL vs. CTRL); °°°° *p* < 0.0001 (PA vs. CHOL).

The mild stem cell death induced by cholesterol in ZL4-SC is accompanied by a nearly 400-fold increase in cellular fat droplets (Figure 3, log scale), stained by Oil Red O over a negligible level in the control. This effect of cholesterol was much higher than what we reported for the dyslipidemic OZ serum [8], but fat infiltration by palmitate was much less pronounced and non-significant.

**Figure 4:**
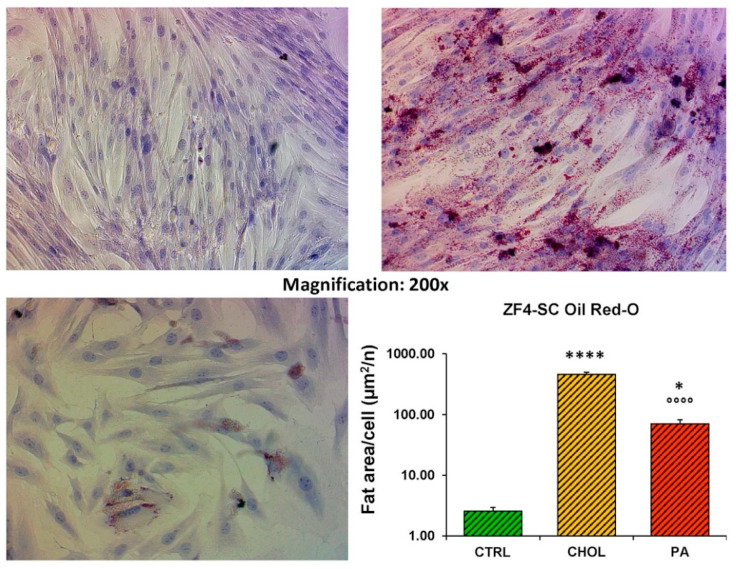
The ZF4-SC were similarly affected as ZL4-SC in the level of fat infiltration by both cholesterol and Na palmitate. For description see legend to Figure 3. **** *p* < 0.0001 (CHOL vs. CTRL); °°°° *p* < 0.0001 (PA vs. CHOL); * *p* <0.05 (PA vs. CTRL).

Again, in the case of ZF4-SC (Figure 4, log scale), their sensitivity to cholesterol was similarly high as on ZL4-SC. For palmitate, it was mildly higher than on ZL4-SC, but not significant.

**Figure 5:**
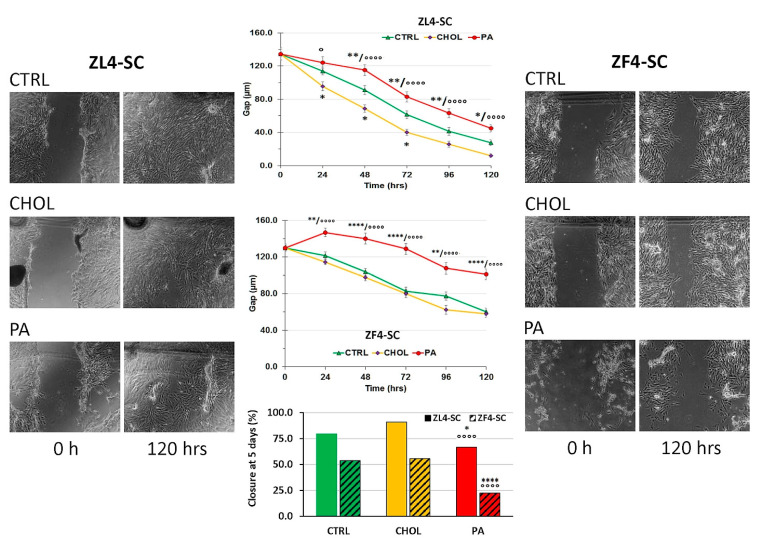
The ZL4-SC were considerably more effective than ZF4-SC on a scratch gap closure healing, and cholesterol improved it, whereas Na palmitate severely impaired it. ZL4-SC and ZF4-SC were incubated separately, as depicted in Figure 1 and Figure 2 but in six-well plates as semi-confluent cultures. The cells were incubated with cholesterol, Na palmitate, or no addition for 4 days. Then a scratch wound was made, and the medium was changed to normal. The progression of the gap closure was monitored and documented for 5 days. Representative micrographs (100×) of ZL4-SC at initiation and completion are shown at the left, and the ones for ZF4-SC at the right. In the middle, the time progression of the healing is shown at the top. * *p* < 0.01 (CHOL vs. CTRL); ** *p* < 0.01; **** *p* < 0.0001 (PA vs. CTRL); ° *p* < 0.05, °°°° *p* < 0.0001 (PA vs. CHOL). The bar graph shows the final, 120 h state. All symbols have uniform meaning.

Next, we verified whether cholesterol impairs stem cells “scratch” closure, as a representation of wound healing in culture [8]. We found (Figure 5) that at 5 days with ZL4-SC it did not (it actually slightly improved it), whereas we had reported that the OZ serum addition had impaired it. However, Na palmitate partially resembled the serum effects, although more moderately. Cholesterol was also protective on ZF4-SC for proliferation and closure (that was slower than ZL4-SC without addition). In turn, palmitate severely impaired it, albeit not as drastically as ZF serum had done [8].

**Figure 6:**
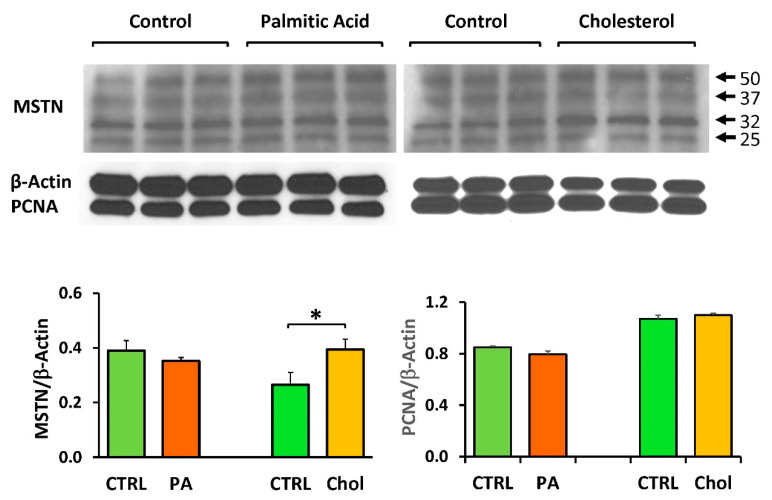
ZF4-SC were only mildly induced by cholesterol, or not by Na palmitate, to overexpress myostatin, a lipofibrotic effector, and not affected for the expression of PCNA, a proliferation mediator. ZF4-SC were incubated in triplicate, as in Figure 5, with or without cholesterol or Na palmitate addition, but until day 4 close to confluence. Then cells were collected for Western blot analysis, probing sequentially with antibodies against myostatin, *β*-actin as a housekeeping protein, and PCNA, followed by the respective secondary antibodies and luminol. The bands for myostatin (MSTN) (50, 32, and 25 kDa) are shown on top. The ones for *β*-actin (42 kDa) and PCNA (36 kDa) from the re-probing and re-exposures are below them. At the bottom: densitometry graphs for MSTN and PCNA, corrected by *β-*actin.; * *p* < 0.05 (CHOL vs. CTRL).

Myostatin overexpression, detected by Western blot, is the feature that characterized the long-term in vivo exposure of MDSC male cells to the T2D/O milieu [5,6] and the in vitro exposure to dyslipidemic serum of both MDSC and the female ZF4-SC [7,8]. It was also investigated here for the lipid factors acting only on ZF4-SC, and not ZL4-SC. The ZF4-SC selection was made to simplify the approach, since they are isolated from the T2D/O rat model, and continued for all successive assays. Beta-actin was used as a housekeeping gene. Figure 6, left panel, shows that cholesterol did stimulate the expression of the main 50 kDa band (and the ancillary 32 and 25 kDa bands), but much less than what we had reported for serum [7], and palmitate did not. The right panel shows that none of both factors stimulated the expression of the proliferation factor PCNA.

**Figure 7:**
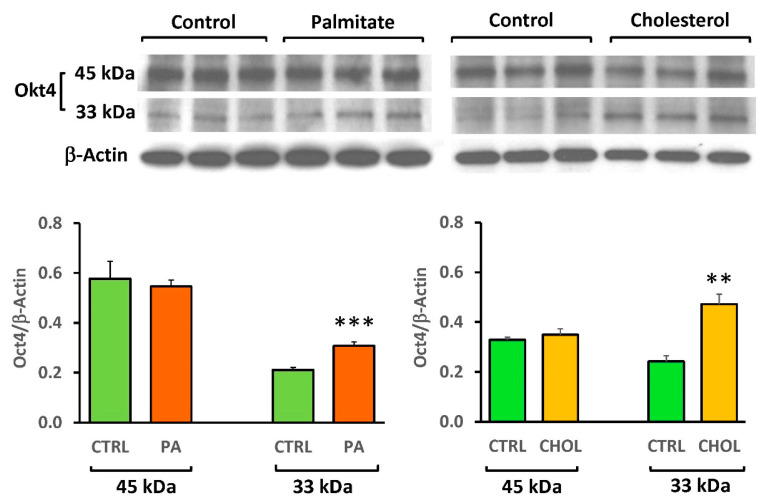
ZF4-SC were not affected by cholesterol or Na palmitate for the expression of the true stemness related form of Oct4, a key stemness factor. ZF4 stem cells were incubated, as on Figure 6, with or without cholesterol or Na palmitate addition. Western blot analysis was conducted as on Figure 6, probing sequentially with antibodies against Oct4 and *β*-actin, followed by the respective secondary antibodies and luminol. The bands for Oct4 (45 kDa, as the true stemness form, and 33 kDa for the stemness unrelated form) are shown on top, and below the ones for β-actin (42 kDa) from the re-probing and re-exposures are below them. At the bottom: densitometry graphs for Oct4, corrected by *β*-actin. *** *p* < 0.001 (PA vs. CTRL); ** *p* < 0.01 (CHOL vs. CTRL).

The potential direct damage in vitro of ZF4-SC stemness by lipid factors was studied as an initial approach by determining by Western blot their effects on the expression of the key stem cell factor octamer-binding transcription factor 4 (Oct 4). Its 45 kDa nuclear isoform is considered as the true stemness isoform Oct4A, rather than its cytoplasmic 33 kDa Oct4B isoform [11,12,13]. Figure 7 shows that, contrary to expectations, both cholesterol and palmitate did not downregulate the 45 kDa band, but upregulated the 33 kDa band, thus altering the 45/33 kDa nuclear/cytoplasmic ratio.

Finally, we had previously reported that the miR-GTSs were considerably affected in both the male MDSC exposed in vivo to T2D/O and in vitro to dyslipidemic serum, and also in vitro in the female ZF4-SC exposed to this serum [5,7,8]. These miR-GTS alterations were also found in male MDSC incubated in vitro with cholesterol and palmitate, affecting many myostatin-related individual miRs [7]. Table 1 shows as reference (left columns for refs 7 and 8) all the myostatin-related miRs, previously reported for MDSC as downregulated in vivo over 2-fold (<0.5 as compared to controls) by the T2D/O milieu, and also downregulated in most cases in the female ZF4-SC to <0.5 by dyslipidemic OZ serum, but not by normal LZ serum. In the Current Results section (right two columns), it is shown that all the miRs in the table were even more downregulated in vitro by palmitate, and in all but one by cholesterol, thus resembling the effects of dyslipidemic serum.

Table 2 makes a similar comparison for the other miRs, presumably unrelated to myostatin. Essentially the same situation as on Table 1 occurred with the Current Results (right panel) for palmitate and cholesterol in vitro on ZF4-SC, versus the previously reported data (left panels) for male MDSC in vivo exposed to T2D/O, and female ZF4-SC in vitro exposed to the dyslipidemic serum.

To determine how close the selected concentrations of cholesterol (50 mg/dL) or sodium palmitate (0.5 mM) in our in vitro tests, represent their in vitro levels when ZF4-SC are incubated with the dyslipidemic ZF serum (OS) vs. the normal ZL serum (LS) as in our previous paper [8], cholesterol levels were measured (see Section 4), in the ZF dyslipidemic and ZL normal sera used previously [8], obtained from five rats/group, and expressed as mean ± SEM.

Total cholesterol in mg/dL was 256 ± 32 for the ZF serum vs. 74 ± 17 for the ZL serum, a significant, 3.5-fold difference in total cholesterol content with a *p*-value of < 0.001. Therefore, the 50 mg/dL in vitro values of added pure cholesterol in the current paper are within an acceptable range for the calculated cholesterol from the ZF serum at 5% in vitro (13 mg/dL) considering the variability of its uptake in the presence of the basal fetal bovine serum (FBS) in the medium. Free cholesterol in mg/dL was 38 ± 5.8 vs. 7.5 ± 1.1 (*p*-value of <0.01), and a 5.0-fold higher free cholesterol content for the ZF vs. ZL sera.

In turn, free fatty acids (FFA) were estimated (see Section 4) in the same female sera above, with three rats/group, and expressed as mean ± SEM in palmitate equivalents. Total FFA in mM was for the ZF serum vs. ZL serum: 4.19 ± 0.79 vs. 0.19 ± 0.01 (*p*-value of <0.001), a 22-fold higher total FFA content for the ZF vs. ZL sera. Therefore, the 0.5 mM in vitro value of added palmitate in the current paper is within an acceptable range for the calculated FFA from the ZF serum at 5% in vitro (0.2 mM) considering the FBS limitation. Palmitate is at least 40–60% of these values.

Finally, to determine whether the HDL/LDL ratio could be a more specific factor than the total or free cholesterol levels for defining the prior effects of cholesterol in serum [8], these lipoproteins were extracted from other aliquots of the same ZF and ZL sera assayed above. HDL levels were measured and referred to the measures of total and free cholesterol in these new assays (Table 3). The new ZF and ZL levels resembled the above ones, also with significantly higher ZF/ZL ratios of 2.2 (total) and 2.7 (free).

The ZF/ZL ratios for HDL, and particularly for LD, were significantly higher in both cases but about to the same levels, with a non-significant difference in the HDL/LDL ratio between ZF and ZL This ratio was higher than in human serum. All other comparisons of HDL and LDL, referred to their total cholesterol values between ZF and ZL were non-significant.

## 3. Discussion

Altogether, it is clear that ZF4-SC, obtained from the young, 12 weeks old female T2D/O-FSUI animal model, were slightly more sensitive than their counterpart ZL4-SC, from the aged-matched female normal lean (non-T2D/O-FSUI) animal model, for in vitro damage by short-term exposure to lipid factors. This, in terms of apoptosis and closure of the scratch wound, was less obvious than for fat infiltration.

Our results confirmed what we had reported for these two types of stem cells during their in vitro exposure to dyslipidemic serum [7,8]. Even if the systemic milieu to which ZF4-SC had been exposed in vivo at this young age was rather similar (nearly normoglycemic and presumably normolipidemic) to the one affecting in vivo ZL4-SC, some other factor in the short ZF4-SC exposure in situ in the T2D/O-FSUI rat model could be responsible for their increased sensitivity. It is remarkable that FSUI in this model has been reported as early as 12 weeks of age [9], and ZF4-SC initial damage in vivo may be related.

Therefore, for simplification, this discussion is restricted to the 4–5 days in vitro effects of lipid factors on ZF4-SC. ZL4-SC in the current paper were excluded from the additional assays applied to ZF4-SC. The concentration of in vitro added cholesterol we chose in our various previous reports and here (50 mg/dL) is much lower than the cholesterolemia of 450 mg/dL we had reported for the male OZ rat at 12 and 28 weeks of age, respectively [5]. At this moderate experimental added concentration of cholesterol, the observed doubling of apoptosis was similar to the one we reported in vitro [8] by 5% OZ female dyslipidemic serum. The nearly 200-fold increase in fat droplets induced by cholesterol was also very similar to the one we had reported for this serum.

An important difference that exists with the dyslipidemic serum was that we had reported that it had blocked completely the in vitro scratch closure. Instead, cholesterol here did not affect it at all despite inducing mild apoptosis. Palmitate had a different action on ZF4-SC since, at 0.5 mM, it was the main inhibitor of scratch closure and increased > 5-fold the apoptotic index, but contributed much less to fat infiltration than cholesterol.

In summary, it appears that palmitate was possibly the driver of pro-apoptotic and wound healing inhibition effects of the OZ female dyslipidemic serum on ZF4-SC, while cholesterol was probably the main contributor to the serum induction of fat infiltration. This may be consistent for cholesterol-induced mild overexpression of myostatin, although much lower than with dyslipidemic serum, but it is difficult to reconcile with the lack of cholesterol effect on PCNA.

The lack of effects of both cholesterol and palmitate on the 45 kDa Oct4 is not necessarily an indication of ZF4-SC stemness resistance. Leaving aside that longer incubations may eventually inhibit this isoform protein expression, other key stemness regulator proteins, like Nanog [11], Sox 2 [12], or several others, might be involved and affected, and all this should be investigated. It is interesting that the cytoplasmic Oct4B, virtually negligible in the controls, was moderately upregulated by cholesterol and particularly palmitate, since this occurs in abnormal stem cell differentiation and several types of cancer [14,15,16,17,18]. However, the interpretation of this observation needs corroboration and further study.

The short-term in vitro inhibition found here by both palmitate and cholesterol of myostatin-related miRs in female ZF4-SC, as well as of top expressed non-myostatin related miRs, is in very good agreement with what we had reported for the in vitro effects of the dyslipidemic serum also in ZF4-SC [8]. This also agrees even with the long-term in vivo effects of T2D/O on male MDSC miRs and the short-term in vitro effects of dyslipidemic serum and lipid factors on male MDSC [7]. However, these miR transcriptional effects apparently require some factor present in the serum other than cholesterol or particularly palmitate alone, to induce myostatin protein overexpression.

Collectively the current results on the in vitro effects of cholesterol or palmitate on the female ZF4-SC damage support the interpretation that dyslipidemia is the main factor that triggers it, like previously in the male MDSC damage. However, work needs to be conducted to show what are the in vivo effects on the female ZF4-SC exposed long-term to the evolving T2D/O milieu in the FSUI rat model, reaching 24 weeks of age (mild glycemia and more intense dyslipidemia). Are these ZF4-SC ineffective in repairing in vivo FSUI, when implanted into the myosphincter? If so, this would replicate what we showed in vivo in the male rat counterpart with late T2D/O MDSC, but for the associated erectile dysfunction [5].

On the other hand, it was confirmed here that the female ZF serum we used previously [8] was clearly and significantly enriched in total and free cholesterol, and palmitate, i.e., highly dyslipidemic as compared to the control female ZL serum. This replicates what we had partially reported in the male model [5,7]. It also ratifies the 50 mg/dL cholesterol and 0.5 mM palmitate used here, as approximate equivalents of the incubations with 2.5–10% added rat serum [8]. Alterations in the HDL/LDL ratio in the ZF vs. ZL sera were non-significant, but a progressive age/T2D/O-related unbalance in favor of LDL may play a role in MDSC damage, if it is found conclusively. However, the HDL/LDL ratio in female Zucker lean and obese rats keeps rather higher than in human serum at least in 20–30 weeks old rats [19], and this may be protective against LDL effects.

Future studies to expand the relevance of our in vitro stem cell damage findings may compare the late T2D/O exposed female ZF4-SC (isolated at perhaps 30–40 weeks of age), against the respective early T2D/O exposed counterparts used here, for some damage parameters we studied in vitro. The above would confirm the in vivo T2D/O damage of the male and female rat MDSC/ZF4-SC, to their potential impact on stem cell therapy in the human. It would validate even further the in vitro studies as non-invasive tools to study mechanisms of the damage and follow up therapy.

The gene-GTS and miR-GTS would also have to be defined in vivo in the late T2D/O exposed ZF4-SC (as we reported [7] in the male LD-MDSC). This would give a more precise proof for future use of the miR-GTS as biomarkers for identification of stem cell damage, and their possible replication in serum, to predict their in vivo efficacy in the animal models. Obviously, to exclude that this applies only to rat MDSC/ZF4-SC, other animal stem cells may have to be studied. However, the ultimate verification would be in key human stem cells used in therapy as autografts in diabetic/obese patients [1,2,3,20,21,22,23].

Other than some recent papers [24,25,26,27] exposing flaws of long-exposed human stem cells to the diabetic milieu in patients, it does not seem to be an awareness of the role that stem cell damage may play in their efficacy for therapy and explaining some clinical failures. The situation is even of more concern regarding the potential role of dyslipidemia, or specifically cholesterol or free fatty acids, in inducing this damage that is virtually ignored. In contrast, the noxious inflammatory milieu [28] or the senescence process [29,30] are occasionally mentioned as a risk. Tangential old citations for cholesterol damaging stem cells [31,32], or more recent for palmitate impact on progenitor cells apoptosis [33], do not seem to have yet challenged the implicit dogma of stem cell resistance to the systemic milieu. The same one that does damage differentiated cells, at least in diabetic/obese subjects. Further investigation is obviously required for defining this topic and its relevance to possible interference with human stem cell therapy, at least in vitro for wound healing [34].

In summary, the current study further demonstrates that cholesterol and palmitate in vitro cause substantial damage to the muscle stem cells isolated from not just the male OZ rat model for T2D/O and erectile dysfunction [7], but from the female OZ model for FSUI. Moreover, that these lipid factors are at least partially responsible for the stem cell damage elicited in vitro by the dyslipidemic serum from these aged rats previously reported [8], and more speculatively that they may be responsible for it in vivo. In addition, we confirmed that the miR-GTS are biomarkers of the stem cell damage exerted in vitro by the dyslipidemic serum and lipid factors. These miR-GTS alterations may occur in vivo by the T2D/O milieu, and serve to predict stem cell damage that may impact therapy for FSUI by the exposed cells, either endogenously or upon implantation in other body sites. Additional dyslipidemic serum components that still need to be clarified may also be involved in vivo and mimicked experimentally in vitro.

In turn, future studies on miR-GTS applied for stem cell damage identification, and even for in vitro replenishment of normal exosomes into stem cells as therapy [35,36], may provide more convincing evidence for the clinical translational value of animal and human stem cell damage research. Thus, we propose that the study of these processes may determine whether they affect other stem cells and eventually impact human stem cell therapy.

## 4. Materials and Methods

### 4.1. Stem Cells Isolation 

The female ZF and ZL rats were housed and treated according to The National Institutes of Health guides and used for isolating serum and stem cells under IACUC approval. To isolate the stem cells [8], the muscle tissue was enzymatically dissociated, first with collagenase, and then dispase, after which non-muscle tissue was gently removed under a microscope. The cell suspension was filtered through a Falcon nylon filter (ThermoFisher Scientific, Waltham, MS, USA) and incubated with the following biotinylated antibodies: CD45, CD11b, CD31, and Sca1 (BD Biosciences, San Jose, CA, USA). Streptavidin beads (Milteny Biotec, San Diego, CA, USA) were then added to the cells together with antibodies for integrin-*α*7–phycoerythrin and CD34–Alexa647 (eBioscience, San Diego, CA, USA), followed by magnetic depletion of biotin-positive cells. The CD45-/CD11-/CD31-/Sca1-/CD34+/integrin-*α*7+ population was then enriched twice by flow cytometry (Becton-Dickinson, San Diego, CA, USA).

ZF4-SC and ZL4-SC were cultured in 0.1% gelatin-coated culture flasks in DMEM (4.5 g/L glucose), 10% FBS, 1% non-essential amino acids, 1% Na-Pyruvate (GE Life Sciences, Marlborough, MA, USA), and used in the 10th–15th passage. Reagents were from Gibco Life Technologies (Waltham, MA, USA).

### 4.2. Stem Cells Incubations and Lipid Factors Estimations in Rat Serum

ZF4-SC and ZL4-SC were incubated (initial 40% confluence) for 4 days on collagen-coated six- or 12-well plates, or eight-removable compartment slides [7,8], without addition (control: C, or CTRL) or adding soluble forms of palmitic acid, Na salt, conjugated to albumin (PA) or cholesterol-methyl cyclodextrin (CHOL), both from Sigma-Aldrich (St Louis, MO, USA), to a concentration of 0.5 mM or 50 mg/dL, respectively. Albumin and methyl-cyclodextrin were not used as vehicle controls because when free, they bind some endogenous PA and cholesterol, respectively. Control wells contained no lipid additives. The medium was then discarded, MDSC were washed with PBS and subjected to fixation for cytochemistry, or fresh protein isolation for Western blots, or RNA isolation for gene/miR-GTS [8].

The total and free cholesterol levels present in the ZF and ZL rat sera previously used [8] were determined to relate to the cholesterol added in the current study, using the Cholesterol Luminometric Assay Kit (Promega, Madison, WI, USA). This same kit was applied to measure directly bound HDL and bound LDL (non-HDL) levels in serum extracts. The free fatty acids (FFA) were determined to relate to the Na palmitate added in the current study as an FFA representative using the FFA Quantification Colorimetric/Fluorometric Kit (Biovision, Milipitas, CA, USA), against the kit palmitate control.

### 4.3. Scratch Wound Assay 

Briefly, stem cells treated as above were plated on 12-well plates to generate 100% confluence in 24 h. Then, a scratch was made through the cell monolayer by pressing a 200 µL pipette tip against the bottom of the well. The detached cells and medium were removed, fresh medium was restored, and pictures were taken with a Nikon Eclipse Ti2 inverted microscope (Nikon Instruments Inc., Melville, NY, USA). The cells were left incubating, and at 4, 24, 48, 72, and 120 h, and pictures were taken to follow wound closure. The distance between both sides of the wound was measured, and the time to complete closure was determined [8,34].

### 4.4. Quantitative Cytochemistry

Cells on the 12-well plates were stained with Oil Red O for fat droplets [5,7], and cells on the eight-well slides were stained for TUNEL assays for the apoptotic index [5,6,35], using duplicate wells. Quantitative image analysis of 100–200× magnification micrographs was performed with ImageProPlus 5.1 (MediaCybernetics, Rockville, MD, USA) on as many fields as necessary to cover the wells (10–12 fields/well) [8].

### 4.5. Western Blots

Immuno-detection on the membranes was with primary antibodies (Santa Cruz Biotechnology, Santa Cruz, CA) against myostatin (GDF8/11, mouse monoclonal antibody SC-393335); and housekeeping beta-actin (mouse monoclonal, followed by secondary antibodies: anti-mouse IgG, horseradish peroxidase (HRP)-linked antibody (Cell Signaling Technology, Danvers, MA, USA), or anti-rabbit IgG linked to HRP (Amersham GE, Pittsburgh, PA, USA). Oct4 was estimated by a polyclonal antibody from Biovision catalog 3576, at 1:500; and PCNA with a monoclonal antibody from Millipore MAB424, at 1:2000. Bands were visualized using luminol (SuperSignal West Pico; Chemiluminescent, Pierce, Rockford, IL, USA). For negative controls, the primary antibody was omitted. Densitometric analysis was performed in certain cases, as stated, correcting by the housekeeping proteins [5,7,8].

### 4.6. Global miR-GTS

RNA was isolated from ZF4-SC and ZL4-SC with mirVana^TM^ miRNA isolation kit (Ambion, ThermoFisher, San Diego, CA, USA), determining quality by the Agilent 2100 Bioanalyzer (Agilent Technologies (Dako) Carpinteria, CA, USA). miR content was estimated by Norgen Biotek Corporation (Thorold, ON, Canada) by next-generation sequencing for all miRs listed in the Sanger miRBase Release 18.0. Values were expressed per 10 million reads. Control values with no lipids addition (C) were tabulated for the ZF4-SC, and then in vitro treatment ratios against C were calculated for samples receiving cholesterol or palmitate [8]. Only miR ratios up- or downregulated by at least 2-fold were selected unless stated. To compare them in the female ZF4-SC to the in vivo male MDSC, the previous in vivo ED/LD MDSC ratios [5,7,8], and also the female ZF4-SC with serum vs. C [8] were included as a reference. The ED-MDSC were from 12 weeks old male OZ rats [7], with mild hyperglycemia/dyslipidemia, and moderate overweight, while the LD-MDSC were from aged, 32 weeks old male OZ rats, with moderate hyperglycemia, high dyslipidemia, and morbid obesity. The miR-GTS complete results are in the GEO library.

### 4.7. Statistical Analysis

Statistical Analysis was performed with GraphPad Prism 7.0 (GraphPad Software, San Diego, CA, USA). When applicable, values were expressed as mean ± SEM. The normality distribution of the data was established using the Wilk–Shapiro test. Direct comparisons were performed using two-tailed unpaired *t*-test. Multiple comparisons were analyzed by single-factor ANOVA, followed by post hoc comparisons with the Tukey multiple comparison test.

## Figures and Tables

**Table 1:** Despite the observed mild overexpression of myostatin protein by cholesterol and its lack of induction by Na palmitate in ZF4-SC, the miRs related to myostatin, were also inhibited by the lipid factors. The current results are shown on the right two columns, for RNA isolated from the standard 4 days incubations of ZF4-SC with Na palmitate and cholesterol or no addition, which were assayed for their miR-global transcriptional signatures (miR-GTS). Only individual miRs related to myostatin expression were selected for tabulation, previously arranged by their level of expression on male MDSC exposed in vivo to type 2 diabetes/obesity (T2D/O) [7] on LD-MDSC vs. non T2D/O in ED- MDSC. Blue highlighting for current results shows downregulation < 0.50 against controls. Yellow highlighting is similarly applied to the same miRs previously reported both in male rats in vivo [7] and in vitro with dyslipidemic Obese Zucker (OZ) serum in female rats [8], and now are included as a reference. Green highlighting is for the miRs, which previously were shown to be in agreement with the in vitro results with dyslipidemic serum in female ZF4-SC.

Source	Ref. 7	Ref. 8		Current Results	
**Conditions**	In vivo	In vitro		In vitro	
	+ or −	+ or −		+ or −	
	T2D/O	serum		lipid factors	
**Gender**	Male	Female		Female	
**Cells**	MDSC	ZF4-SC		ZF4-SC	
**Factors**	T2D/O	5% serum (S)		Palmitic acid 0.5 mM	Cholesterol 50 mg/dL
**Data comparison**	LD/ED	OS/C	LS/C	PA/C	CHOL/C
**ID for miR or let**	Ratios to respective ED or C				
**miR-21-5p**	0.16	0.52	1.17	0.08	0.27
**miR-199a-5p**	0.29	0.41	0.86	0.11	0.38
**miR-23a-3p**	0.50	1.11	1.09	0.10	0.27
**miR-199a-3p**	0.25	0.41	0.86	0.10	0.30
**miR-27a-3p**	0.41	0.99	1.08	0.14	0.32
**miR-181a-5p**	0.39	0.54	0.94	0.19	0.38
**miR-30e-5p**	0.42	0.44	1.14	0.25	0.61
**miR-101a-3p**	0.38	1.06	0.97	0.17	0.36
**miR-214-3p**	0.22	0.46	0.98	0.17	0.32
**miR-29a-3p**	0.25	0.59	1.26	0.22	0.44
**miR-101b-3p**	0.09	0.49	1.13	0.16	0.37
**miR-132-3p**	0.02	0.68	0.99	0.27	0.06

**Table 2:** A similar decrease of key miRs unrelated to myostatin by lipid factors was found in ZF4-SC. See Table 1 for the legend explanation, except that here the selected miRs were unrelated to myostatin. Within those selected miRs were the ones most expressed and changed by incubation as reported previously [8]. Purple highlighting shows the only two in vivo, which within the selected miRs were upregulated.

Source	Ref. 7	Ref. 8		Current Results	
**Conditions**	In vivo	In vitro		In vitro	
	+ or −	+ or −		+ or −	
	T2D/O	serum		lipid factors	
**Gender**	Male	Female		Female	
**Cells**	MDSC	ZF4-SC		ZF4-SC	
**Factors**	T2D/O	5% serum (S)		Palmitic acid 0.5 mM	Cholesterol 50 mg/dl
**Data comparison**	LD/ED	OS/C	LS/C	PA/C	CHOL/C
**ID for miR or let**	Ratios to respective ED or C				
**miR-99b-5p**	0.25	0.53	0.89	0.18	0.30
**miR-10a-5p**	0.28	0.66	0.95	0.14	0.37
**miR-100-5p**	0.22	0.63	0.78	0.16	0.39
**miR-99a-5p**	2.65	0.37	0.95	0.22	0.40
**miR-10b-5p**	0.45	0.33	0.92	0.15	0.43
**miR-26a-5p**	0.32	0.99	1.12	0.18	0.39
**let-7f-5p**	0.32	0.89	1.14	0.13	0.30
**miR-221-5p**	0.34	0.59	1.26	0.30	1.22
**let-7i-5p**	0.12	0.86	1.11	0.10	0.33
**miR-148a-3p**	0.25	0.27	0.86	0.15	0.37
**miR-152-3p**	0.33	0.42	0.97	0.09	0.24
**miR-148b-3p**	0.16	0.51	1.06	0.13	0.36
**let-7g-5p**	0.22	1.10	1.08	0.06	0.16
**miR-92a-3p**	0.17	0.59	1.19	0.36	1.00
**miR-342-3p**	0.26	0.22	0.99	0.06	0.23
**miR-212-5p**	0.08	0.87	1.12	0.29	0.43
**miR-10b-3p**	0.10	0.69	1.08	0.19	0.37
**miR-25-3p**	2.20	0.67	0.94	0.25	0.64
**miR-362-5p**	0.20	0.36	1.56	0.15	0.73
**miR-31a-5p**	0.29	1.40	1.24	0.08	0.20
**miR-99b-5p**	0.25	0.53	0.89	0.18	0.30

**Table 3:** The total and free cholesterol, as well as the HDL and LDL contents in the female ZF serum, are significantly higher than in the ZL serum, but the HDL/LDL ratios are not significantly different (*n* = 5 per serum type in duplicates, all values in mg/dL, NS: non-significant).

	ZL Serum	ZF Serum	ZF/ZL	*p* Value
Free Cholesterol	29.5 +/− 2.2	77.7 +/− 7.4	2.63	<0.0001
Total Cholesterol	98.1 +/− 3.8	214.4 +/− 3.8	2.18	<0.0001
HDL	43.5 +/− 2.7	75.3 +/− 3.3	1.73	<0.0001
Non-HDL	25.2 +/− 1.7	58.4 +/− 6.2	2.32	<0.0001
**Ratios**				
Total/Free	3.44 +/− 0.2	3.04 +/− 0.4	0.88	NS
Total/HDL	2.57 +/− 0.6	2.87 +/− 0.1	1.12	NS
Total/non-HDL	4.10 +/− 0.4	3.90 +/− 0.3	0.95	NS
HDL/non-HDL	1.32 +/− 0.2	1.39 +/− 0.1	1.05	NS

## Abbreviations

C or CTRL	incubation controls without any addition
CHOL	cholesterol
ED-MDSC	early diabetes MDSC from male 12 weeks old Obese Zucker (OF) rats (Crl:ZUC Leprfa) with mild hyperglycemia/dyslipidemia and moderate overweight
Gene-GTS	gene global transcriptional signature
LD-MDSC	late diabetes MDSC from male 32 weeks old ZF rats, with moderate hyperglycemia, high dyslipidemia, and morbid obesity
MDSC	muscle-derived stem cells
miR	microRNA
miR-GTS	miR global transcriptional signature
PA	palmitic acid/Na palmitate
T2D/O	type 2 diabetes mellitus/obesity
ZF	Zucker obese fatty rats
ZF4-SC	stem cells from 12 weeks old female ZF rats
ZL	Zucker lean rats
ZL4-SC	stem cells from 12 weeks old female ZL rats
